# LncRNA CALML3-AS1 suppresses papillary thyroid cancer progression via sponging miR-20a-5p/RBM38 axis

**DOI:** 10.1186/s12885-022-09360-3

**Published:** 2022-03-29

**Authors:** Xiaozhou Zhang, Xiaojian Zhang, Qian Jia, Hongqiang Li, Runsheng Ma, Guang Yang, Fengyan Yin, Nannan Jiang, Detao Yin

**Affiliations:** 1 Department of thyroid surgery, The First Affiliated Hospital of Zhengzhou University, No. 1 Jianshe East Road, Zhengzhou, 450000 Henan Province China; 2 Department of thyroid surgery, Taian City Central Hospital, No. 29 Longtan Road, Tai’an, 271000 Shandong Province China; 3 Pharmacy Department, The Second Affiliated Hospital of Shandong First Medical University, No. 706 Taishan Street, Tai’an, 271000 Shandong Province China

**Keywords:** Papillary thyroid cancer, lncRNA, lncRNA CALML3-AS1, miR-20a-5p, RBM38

## Abstract

**Background:**

The incidence and mortality of thyroid cancer (TC) has been steadily rising in the past decades. It is imperative to have a better understanding of the molecular mechanisms underlying TC development and identify novel therapeutic targets. This study characterized the role of lncRNA CALML3-AS1 (CALML3-AS1) in the development of papillary thyroid cancer (PTC).

**Method:**

Related mRNAs expression were validated in the tumor and adjacent normal tissues from 52 PTC patients and PTC cell lines by qRT-PCR. Expression of RBM38 was detected by Western blot. We have also conducted CCK-8 and colony formation assays were used to detect the effect of CALML3-AS1 on cell proliferation, Transwell assay was utilized to evaluate cell migration and invasion, apoptosis detected by flow cytometry assay, RNA pull-down and luciferase assays were performed to validate gene predictions.

**Results:**

The results indicated that the expression of both CALML3A-S1 and RBM38 were significantly downregulated in PTC tissues (*p* < 0.01), while the expression of miR-20a-5p was increased in PTC (*p* < 0.01). Functionally, CALML3-AS1 overexpression inhibited PTC cell proliferation in vitro and in vivo. Mechanistically, CALML 3-AS1 sponged miR-20a-5p, which in turn leads to the suppression of RBM38 expression and PTC progression.

**Conclusions:**

CALML3-AS1 functions as a ceRNA for miR-20a-5p in the regulation of the expression of RBM38 in PTC. Higher level of CALML3-AS1 serves as a good prognostic indicator of survival in PTC patients. Targeting CALML3-AS1/ miR-20a-5p/RBM38 axis may represent a novel therapeutic strategy in the treatment of PTC.

**Supplementary Information:**

The online version contains supplementary material available at 10.1186/s12885-022-09360-3.

## Introduction

The past decades have seen steady increase in the incidence and mortality of thyroid cancer (TC). Because thyroid glands secrete physiologically essential hormones such as thyroxine, TC threatens human health and development [1–4]. The development of TC differs in regard to different pathological classifications [5]. Generally, TC originates from two different cell types: follicular cells and parafollicular cells. More than 90% of TCs develop from follicular cells, the thyroid epithelial cells that uptake iodine from blood and synthesize thyroid hormones [6, 7]. Among follicular TCs, differentiated thyroid cancer (DTC) is the most common type, accounting for more than 95% of TCs [8]. Furthermore, about 80% DTCs are papillary thyroid cancers (PTCs), which are the most common subtype of TC [9–12].

Although most PTCs are in benign condition, their biological characteristics can be diverse, spanning from non-progressive/extremely indolent lesions to aggressive metastatic tumors [13], Importantly the late-stage survival rate for PTC is less than 59% [14]. Traditional thyroid surgery may cause permanent damage to the recurrent laryngeal nerve, often resulting in lifelong hypoparathyroidism and thyroxine replacement therapy after the entire thyroidectomy [15]. Therefore, a better understanding of the underlying pathogenic mechanisms and hence identification of novel therapeutic targets and strategies is critical for the development of future effective therapies of PTC.

In recent years, multiple studies showed that lncRNAs can act as important oncogenes or tumor suppressors in the development of many cancers [7, 16], such as in thyroid cancer [17–19]. Research shows that, lncRNAs sometimes serve as ‘sponges’ to titrate microRNA (miRNA). CALML3-AS1 is a newly discovered lncRNA and is transcribed from the CALML3 gene on 10p15.1. However, the role of CALML3-AS1 in PTC remains unknown. In this study, we elucidate the role of CALML3-AS1 in PTC development. Our findings that CALML3-AS1 serves as a tumor suppressor for PTC via sponging miR-20a-5p/RBM38 axis which would guide new therapy development in PTC treatment.

## Materials and methods

### Tissue samples

The tumor and adjacent noncancerous tissues were collected from 52 cases of patients in Taian City Central Hospital who received thyroidectomy but did not receive radiotherapy or chemotherapy before surgical operation. The samples were snap-frozen and stored at − 80 °C for further analysis. All the clinical procedures were in accordance with the declaration of Helsinki and written informed consents were collected from all the participants of the study. All the protocols were reviewed and approved by the committee for human experimentations, Taian City Central Hospital, China.

### RNA extraction and quantitative real-time polymerase chain reaction (qRT-PCR)

Total RNA was isolated from PTC cell lines with Trizol (Invitrogen, USA), and reverse transcribed using PrimeScriptTM RTMaster Mix (TaKaRa, Janpan). qRT-PCR was operated on Lightcycler 480 instrument (Roche, Basel, Switzerland) using SYBR Premix Ex Taq II (TaKaRa, Janpan). In this experiment, GAPDH was an internal control and the 2^−ΔΔCt^ method was used to calculate the mRNA expression levels. All primers were purchased from Invitrogen with sequence as follows: GAPDH.

F: 5′-AAGGTGAAGGTCGGAGTCA-3′;

R: 5′-GGAAGATGGTGATGGGATTT-3′.

LncRNA CALML3-AS1:

F: 5′-TGCAGTGTCACTCTGGAAGC-3′;

R: 5′-CACTGTCTCAGGCCAGGTTT-3′;

MiR-20a-5p:

F: 5′-ATTTCACGAATATCACGT-3′;

R: 5′-CAGTGCGTGTCGTGGAGT-3′;

RBM38:

F: 5′-CTGCCGTACCACACTACCG-3′;

R: 5′-ATGATGGGGTTCGGGTCTTTG-3′;

Si-RBM38–1#:

F: 5′-AAAUAGUUCUCAGUCAUUCUU-3′;

R: 5′-GAAUGACUGAGAACUAUUUAA-3′;

Si-RBM38–2#:

F: 5′-UUAACUUCUUGCUUUCAGGUU-3′;

R: 5′-CCUGAAAGCAAGAAGUUAAUG-3′.

### In Silico analysis of miRNA and gene expression levels

Analysis of the potential binding miRNA partners of CALML3-AS1 was done using the algorithm Lnctar (URL: http://www.cuilab.cn/lnctar). Starbase (URL: http://starbase.sysu.edu.cn/) analysis was used to search the mRNA downstream target of miR-20a-5p.

### Cell culture, plasmids and transfection

PTC cell lines, consist of BHP5–16, BHP2–7, BCPAP and K1, respectively, together with the normal thyroid epithelial cell line (Nthy-ori 3–1) were purchased and obtained from the Shanghai Institute of Chinese Academy of Sciences Cell Collection. Cell lines were inoculated in Dulbecco’s modified Eagle’s medium (DMEM, Gibco) containing 10% FBS and 1% penicillin/streptomycin solution. All cell lines were cultured at 37 °C and under 5% CO_2_. For CALML3-AS1 overexpression, the full-length sequence of CALML3-AS1 was cloned into pcDNA3.1 (Invitrogen, CA, USA) plasmid to generate pcDNA3.1-CALML3-AS1 (CALML3-AS1 OE). To silence RBM38, Small interfering RNAs (siRNAs) against RBM38, miR-20a-5p mimics, and their EV empty vectors were purchased from Shanghai Biotend Biotechnology Co, Ltd. (Shanghai, China). And the siRNAs sequence is: 1#: 5′-CTATGACCAGTACCCATACG-3′, 2#: 5′-GCAGAAGGACACCACGTTCA-3′ and si-NC: 5′-CAACAAGATGAAGAGCACCAA-3′. Transfections were done with Lipofectamine 2000 following the manufacturer’s instruction. qRT-PCR was conducted to validate transfection efficiency of stable expression cell line.

### Cell proliferation assay

The Cell Counting Kit-8 (CCK-8) assay was used for detecting the proliferation of PTC cells. Briefly, 2 × 10^3^ cells were added into the 96-well plates and cultured for 0, 24, 48 and 72 h, respectively. Then, 10 mL CCK-8 solution (YEASENBio, Shanghai, China) was added and incubated in the dark at 37 °C for another 2 h. The number of viable cells was evaluated by measuring the absorbance at 450 nm.

### Colony formation assay

Five hundred cells of BCPAP and K1 were seeded into 6-well plates and incubated for 2 weeks with DMEM containing 10% FBS. After that, 4% paraformaldehyde was fixed and 0.4% crystal violet was stained (Beyotime, China) for 30 min. The colonies were counted with an inverted microscope.

### Transwell migration and invasion assay

Migration and invasion of PTC cells was determined by the transwell assay. After transfection for 48 h, 2 × 10^5^ cells were seeded into the upper chamber (8.0 μm pore size; Corning, USA) with a porous membrane with Matrigel solution (BD, USA) in serum-free DMEM medium, while the lower chamber was inserted into a 12-well filled with 600 mL medium added with 10% FBS. After 48 h, the non-migrated and non-invaded cells on the upper side of the chamber were rinsed off, while the migrated and invaded cells were fixed with 4% formaldehyde and stained with 0.1% crystal violet. Finally, the cells were imaged and counted.

### Flow cytometry

Flow cytometry was performed to analyze the apoptosis of indicated cells. Annexin V-fluorescein isothiocyanate (FITC) apoptosis detection kit (Keygen, Nanjing, China) was used,following the manufacturer’s protocol. CellQuest software version 0.9.3.1 (BD Biosciences) was used to calculate the flow cytometry results.

### In vivo xenograft experiments

Six weeks old BALB/c nude female mice were used to conduct xenograft experiments. Briefly, a density of 4 × 10^5^ transfected cells were subcutaneously injected into the flank of BALB/c nude mice. The mice were maintained under SPF condition throughout the experiment. The tumor volume was checked and measured regularly and calculated based on the following formula: Tumor volume (mm^3^) = (width) × (height)^2^/2. The mice were finally sacrificed after 30 days and the tumor tissue was harvested and weighed. All the experimental protocols were approved by the animal ethical committee of Taian City Central Hospital and were in accordance with the international guidelines for animal experimentation.

### Dual-luciferase reporter assay

To validate the interaction between miR-20a-5p, CALML3-AS1 and RBM38, we have amplified the fragment of CALML3-AS1 and 3′-UTR regions of RBM38 and cloned them to a downstream region of the Renilla psiCHECK2 vector (Promega, Madison, WI), which were named CALML3-AS1 wild type (CALML3-AS1-wt) and RBM38 wild type (RBM38-wt). Mutations were made in the sequence in 3′-UTR of CALML3-AS1 and RBM38 to disrupt binding (CALML3-AS1-mut and RBM38-mut). Then, the plasmids were transfected with miR-20a-5p mimics or empty vector as negative control, respectively. After 48 h, we used Dual-luciferase Reporter assay system (Yeasen, Shanghai, China) to measure the relative luciferase activity.

### RNA pull-down assay

CALML3-AS1 and Oligo (specifically bind to mRNA) were biotinylated to create bio-CALML3-AS1 and bio-Oligo by GenePharma Company (Shanghai, China), which were subsequently transfected into BCPAP and K1 cells for 48 h. The cell lysates were incubated with Dynabeads M-280 Streptavidin (Invitrogen, USA) for 10 min for pull down of biotinylated RNA or Oligo. Finally, the cells were washed by buffer and following detected RNA enrichment by qRT-PCR.

### Western blot analysis

Briefly, BCPAP and K1 cells were harvested and lysed with radioimmunoprecipitation assay (RIPA) buffer (Solarbio, Beijing, China). Total protein concentration was determined with the BCA Assay (Beyotime, China). The protein (30 μg per lane) was separated on a 10% SDS-polyacrylamide gel and transferred to a polyvinylidene fluoride membrane (Millipore, Billerica, MA, USA). The primary antibodies of GAPDH (5174 T, Cell Signaling Technology Pathways, CST) and RBM38 (sc-365,898, Santa Cruz Biotechnology) were diluted 1:1000 and incubated at 4 °C overnight. The secondary antibodies were selected according to each primary antibody’s instructions. The membranes were washed three times with TBST and visualized using SuperSignal West Dura Extended Duration Substrate following the manufacturer’s protocol.

### Statistical analysis

Statistical analyses were performed using Graphpad Prism statistical software (Version 6.0; La Jolla, CA, USA). Results are shown as mean ± SD. The significance of difference was evaluated with Student’s t-test in two groups. Correlations among CALML3-AS1, miR-20a-5p and RBM38 were analyzed with Pearson correlation analysis. *p* values less than 0.05 were considered significant (^#^*p* < 0.05; ^##^*p* < 0.01, *p < 0.05; **p < 0.01).

## Results

### Low expression of CALML3-AS1 is correlated with poor prognosis in PTC patients

**Fig. 1:**
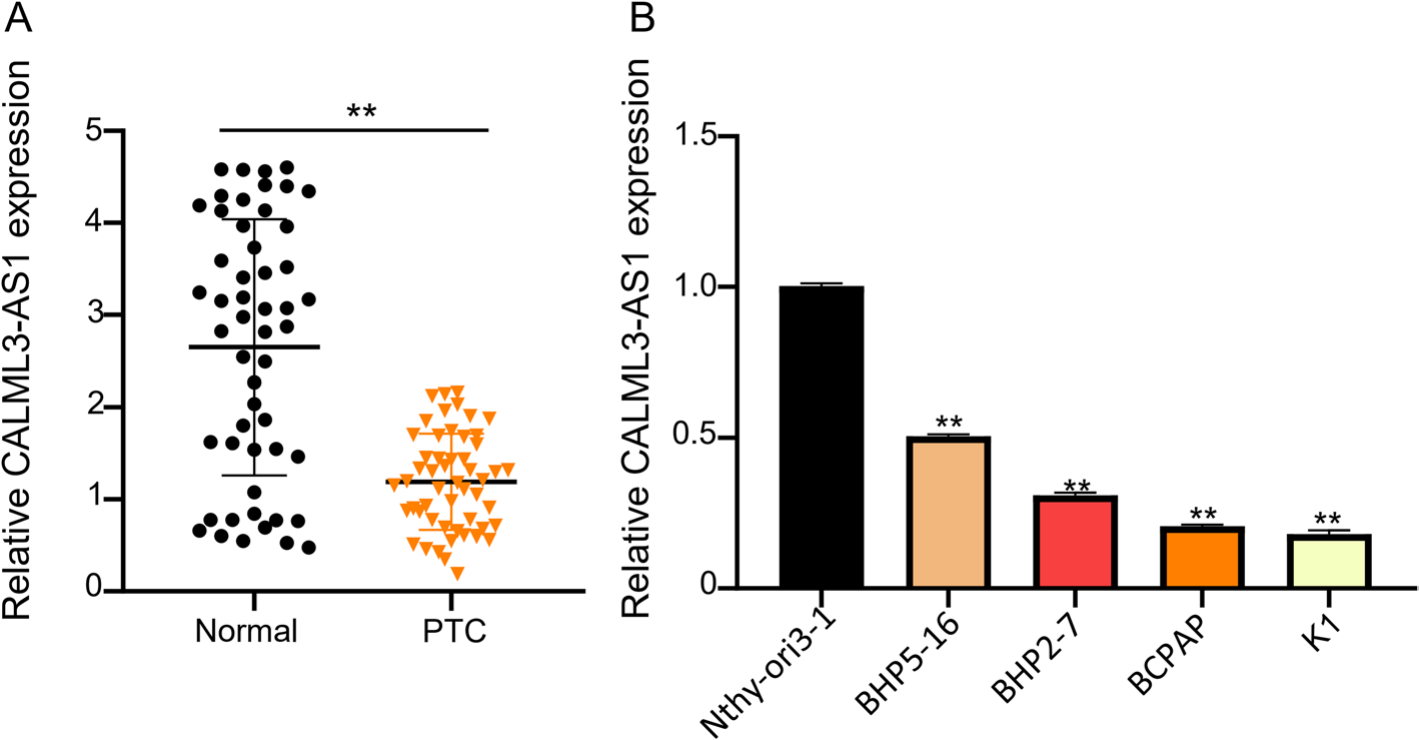
Downregulation of CALML3-AS1 predicted favorable prognosis of PTC patients. **A**, **B**. The expression level of CALML3-AS1 was analyzed using qRT-PCR in 52 cases of PTC tissues and and cell lines (four PTC cell lines and one normal cell line). C. Survival curve was evaluated with Kaplan–Meier method. *P < 0.05, **P < 0.01

To investigate the clinical significance of CALML3-AS1 expression in PTC patients, we collected the tumor and adjacent normal tissues from 52 cases of PTC patients and applied a qRT-PCR analysis to examine the expression of CALML3-AS1. Compared with adjacent normal tissues, CALML3-AS1 expression was significantly reduced in PTC tissues (Fig. 1A, *p* < 0.01). We further confirmed this result by comparing the CALML3-AS1 expression between the different PTC cell lines and the normal human thyroid epithelial cells. As shown in Fig. 1B, the expression of CALML3-AS1 was dramatically down-regulated in all tested PTC cells, compared with Nthy-ori3–1 (*p* < 0.01). The median value of CALML3-AS1 expression in PTC was used as the cut-off value to divide PTC patients into two groups: low level of CALML3-AS1 (*n* = 26) and a high level (*n* = 26). Table 1 evaluated the relationship between CALML3-AS1 expression and clinicopathological data of PTC. The result revealed that the expression level of CALML3-AS1 significantly negatively correlated with tumor sizes (*p* = 0.002) and lymph node metastasis (*p* = 0.026), but not with other clinical factors in PTC patients.

**Table 1:** The clinical data of the patients with PTC

Variable	CALML3-AS1 expression		*P* value
	High (*n* = 26)	Low (*n* = 26)	
Age			
< 55	15	12	0.405
≥55	11	14	
Gender			
Male	13	17	0.262
Female	13	9	
Extra thyroidal extension			
Negative	16	15	0.777
Positive	10	11	
Tumor size (cm)			
≤1	17	6	0.002^*^
> 1	9	20	
TNM stage			
I/II	21	19	0.510
III/IV	5	7	
Lymph node metastasis			
Negative	18	10	0.026^*^
Positive	8	16	

*p < 0.05

### Overexpression of CALML3-AS1 inhibits proliferation, migration and invasion and promotes apoptosis of PTC

**Fig. 2:**
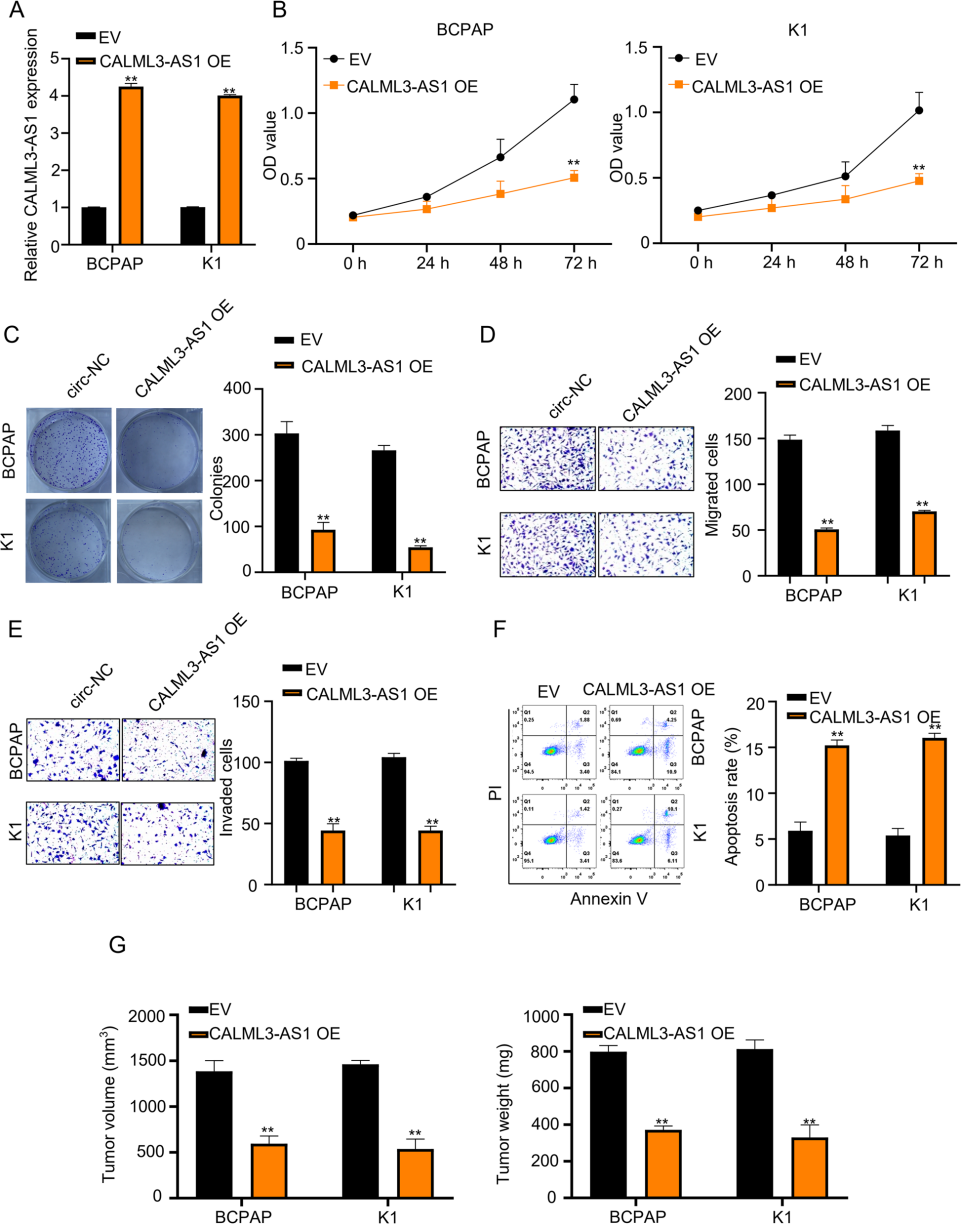
Overexperssion of CALML3-AS1 inhibited cell proliferation, migration and invasion and promoted cell apoptosis. **A**. CALML3-AS1 was overexpressed in BCPAP and K1 cells by cloning into pcDNA3.1, empty vector was used as a control. **B**, **C**. CCK-8 and colony formation assays were used to measure the proliferation ability of BCPAP and K1 cells with EV or CALML3-AS1-OE. **D**, **E**. Transwell assays were used to determine the effects of CALML3-AS1 on PTC cell migration and invasion. **F**. Apoptosis of PTC cells with CALML3-AS1-OE was evaluated with flow cytometry analysis. **G**. Xenograft model was conducted to study the effect of CALML3-AS1-OE on the tumor growth. *P < 0.05, **P < 0.01

To investigate the function of CALML3-AS1 in PTC progression, we overexpressed CALML3-AS1 (CALML3-AS1 OE) in BCPAP and K1, which are CALML3-AS1-low expressed cell lines (Fig. 2A). CCK-8 results revealed that the overexpression of CALML3-AS1 markedly inhibited BCPAP and K1 cell proliferation in a time-dependent manner (Fig. 2B, *p* < 0.01), which was consistent with the results of colony formation assay (Fig. 2C, *p* < 0.01). In addition, we detected the migration and invasion ability of PTC cells after overexpression of CALML3-AS1 (Fig. 2D, E). Transwell assay results showed that overexpressed CALML3-AS1 dramatically suppressed the migration and invasion of BCPAP and K1 cells. Next, we used flow cytometry analysis to examine apoptosis and the results show that apoptosis rate was enhanced in BCPAP and K1 cells after CALML3-AS1 overexpression (Fig. 2F, *P* < 0.01).

To monitor the effect of CALML3-AS1 on the tumor growth in vivo, CALML3-AS1-overexpessing and control BCPAP and K1 cells were injected into the nude mice. The results confirmed that overexpression of CALML3-AS1 efficiently suppressed tumor growth in vivo, resulting in a reduction in tumor weight and volume (Fig. 2G).

### miR-20a-5p is a target of CALML3-AS1

**Fig. 3:**
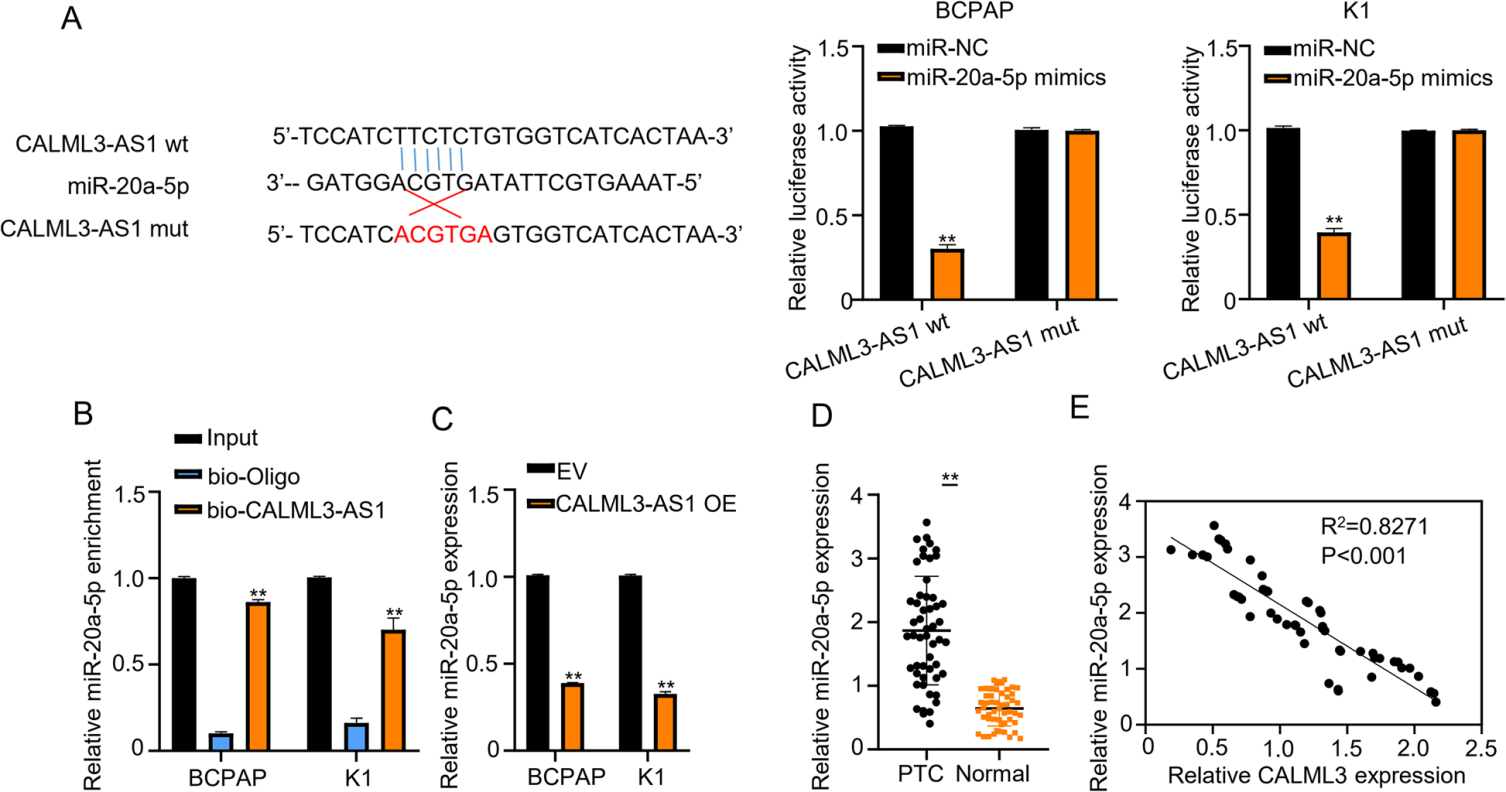
miR-20a-5p is a target of CALML3-AS1. **A**. The binding sites between wild type CALML3-AS1 or mutated CALML3-AS1 and miR-20a-5p were predicted using LncTar analysis. Luciferase reporter assay was carried out in BCPAP and K1 cells to confirm the combination between CALML3-AS1 and miR-20a-5p. **B**. RNA pull-down assays were used to determine the interaction between CALML3-AS1 and miR-20a-5p. **C**. Relative miR-20a-5p expression while CALML3-AS1 overexpression in BCPAP and K1 cells. **D**. The relative miR-20a-5p expression was tested in both BCPAP and K1 cells using qRT-PCR. **E**. Pearson correlation analysis between miR-20a-5p and CALML3-AS1 expressions in 52 cases of PTC tissues. *P < 0.05, **P < 0.01

Previous studies suggest that lncRNAs can act as sponges for certain miRNAs and regulate tumor development through miRNAs. Therefore, we speculated that CALML3-AS1 acts as a competing endogenous RNA (ceRNA) to exert a tumor suppressor effect in PTC cells. To this end, we first analyzed the potential binding miRNA partners of CALML3-AS1 using the LncTar (Fig. 3A) and found that miR-20a-5p potentially interacts with CALML3-AS1. Luciferase reporter assay analysis confirmed that the ectopic expression of miR-20a-5p mimics inhibited the luciferase activity of CALML3-AS1 reporter vector in BCPAP and K1 cells (*p* < 0.01) and such inhibition was impaired by the mutations in the CALML3-AS1 binding motif (Fig. 3A). In addition, RNA pull-down assays determined that the miR-20a-5p expression was more enriched on biotin-labeled CALML3-AS1 probes than unlabeled probe (Fig. 3B). Moreover, overexpression of CALML3-AS1 suppressed miR-20a-5p exppression in BCPAP and K1 cells (Fig. 3C). Furthermore, miR-20a-5p was significantly upregulated in the tumor tissues in 52 cases of human PTC patients, as compared with the adjacent normal tissues and in PTC cell lines, as compared with Nthy-ori 3–1 cells (Fig. 3D). Accordingly, we found a strong inverse correlation between the levels of CALML3-AS1 and miR-20a-5p (Fig. 3E, *p* < 0.001). All these results indicated that CALML3-AS1 directly interacts with miR-20a-5p and inhibits miR-20a-5p expression.

### The downstream target of CALML3-AS1 is RBM38

**Fig. 4:**
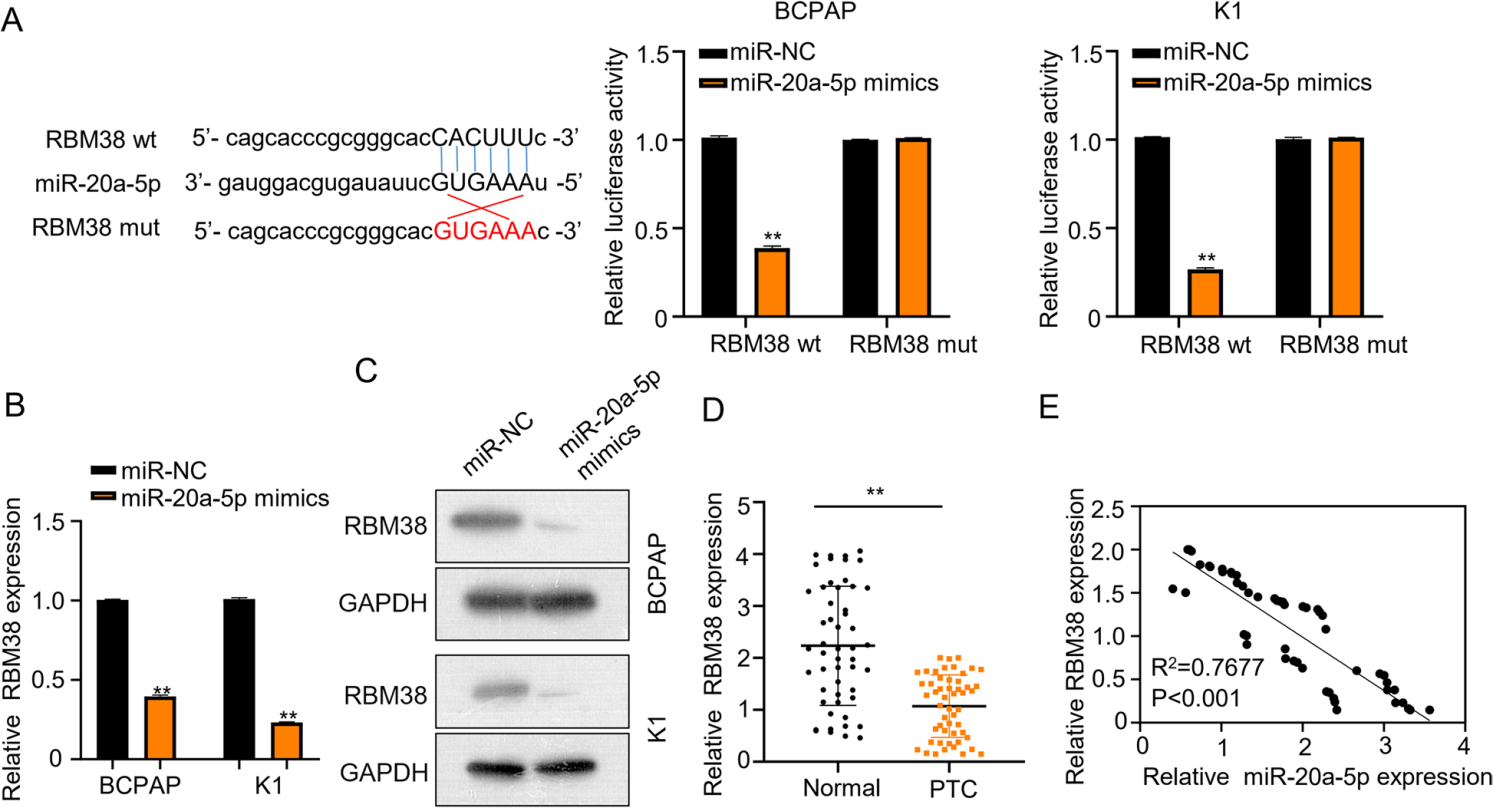
RMB38 is a downstream target of miR-20a-5p. **A**. The binding sites between wild type RMB38 or mutated RMB38 and miR-20a-5p were predicted using Starbase analysis and luciferase reporter assay was carried out in BCPAP and K1 cells to confirm the interaction between RMB38 and miR-20a-5p. **B**. Relative mRNA levels of RMB38 in miR-20a-5p-overexpressing BCPAP and K1 cells were measured using qRT-PCR. **C**. The expression of RMB38 in BCPAP and K1 cells, which overexpress miR-NC or miR-873-5p mimics, was detected by western blot assay. **D**. The relative RMB38 expression was examined in both BCPAP and K1 cells using qRT-PCR. **E**. Pearson correlation analysis between miR-20a-5p and RMB38 expression in 52 cases of PTC tissues. *P < 0.05, **P < 0.01

In order to verify that CALML3-AS1 acts as ceRNA, we searched for the mRNA as the downstream target of miR-20a-5p. Through Starbase analysis, miR-20a-5p was found to bind to the 3′-UTR region of RBM38. Luciferase reporter assays confirmed that the luciferase activity of RBM38-wt) was markedly reduced by miR-20a-5p mimics in BCPAP and K1 (*P* < 0.01) cells. However, the inhibitory effect of miR-20a-5p mimics was completely abolished by the mutations introduced into the binding site of RBM38 (Fig. 4A). Furthermore, qPCR showed that miR-20a-5p mimics, i.e. overexpression, efficiently inhibited the expression of RBM38 in BCPAP and K1 cells, as compared with the normal expression of miR-20a-5p (miR-NC) (Fig. 4B). Western blot analysis also confirmed that, when miR-20a-5p is overexpressed, the expression level of RBM38 protein decreased in BCPAP and K1 cells (Fig. 4C). Similarly, the expression of RBM38 in PTC tissues was significantly downregulated, as compared to normal thyroid epithelial cells (Fig. 4D, *p* < 0.01). At last, the negative correlation between miR-20a-5p and RBM38 were analyzed (Fig. 4E, *p* < 0.001). Together, these results demonstrate that the downstream target of CALML3-AS1 is RBM38.

### The restoration of RBM38 override the effect of CALML3-AS1 overexpression

**Fig. 5:**
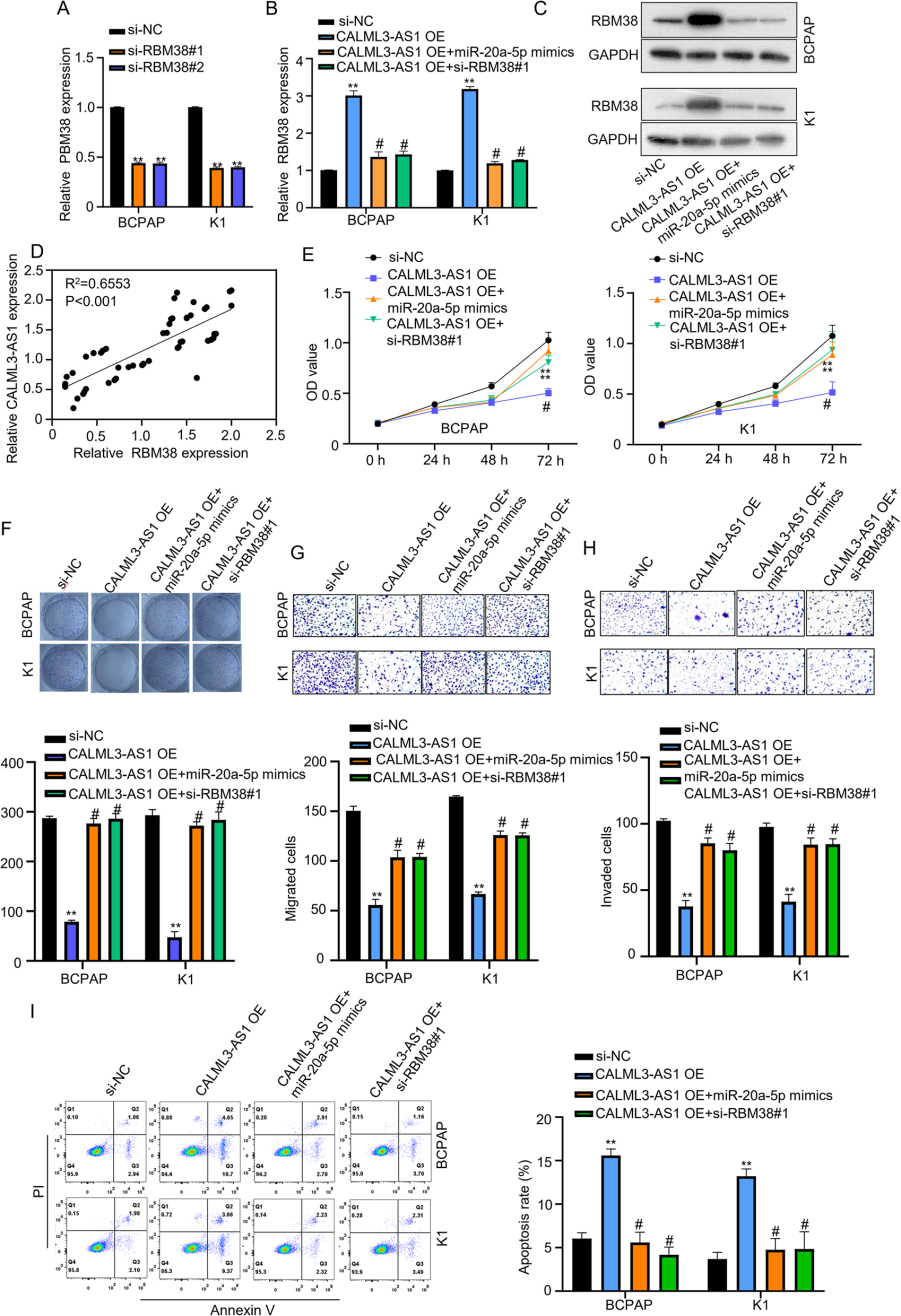
CALML3-AS1 acts as a suppressor via regulation of miR-20a-5p/RMB38 axis. **A**. Relative expression of RMB38 in BCPAP and K1 cells with RBM38 silenced (si-NC, si-RBM38#1, si-RBM38#2). **B**. Relative expression levels of RBM38 with CALML3-AS1-OE, or both CALML3-AS1 and miR-20a-5p, both hsa_circ_0000069-OE and RBM38 knockdown in BCPAP and K1 cells were measured using qRT-PCR. **C.** The expression of RMB38 in BCPAP and K1 cells with si-NC, CALML3-AS1 OE, CALML3-AS1 OE + miR-20a-5p mimics and CALML3-AS1 OE + si-RBM38#1 was detected by western blot assay. **D**. Pearson correlation analysis between CALML3-AS1 and RMB38 expressions in 52 cases of PTC tissues. **E**. **F**. CCK-8 and colony formation assays were used to measure the proliferation ability of BCPAP and K1 cells with the above four groups. **G**. **H**. Transwell assays were used to determine the effects of four groups on PTC cell migration and invasion. **I**. Apoptosis of four groups on PTC cells was examined with flow cytometry analysis. *P < 0.05, **P < 0.01

In order to verify CALML3-AS1 inhibits PTC through miR-20a-5p/RBM38 axis, we first knocked down RBM38 expression (si-RBM38#1 and si-RBM38#2). qRT-PCR analysis showed that, compared with control siRNA, si-RBM38#1 and si-RBM38#2 effectively knocked down RBM38 and the knockdown efficiency was greater than 50% (Fig. 5A, *p* < 0.01). Accordingly, the level of RBM38 was increased when CALML3-AS1 was overexpressed in PTC cells, However, when co-transfected with miR-20a-5p or si-RBM38 pool, the rise in CALML3-AS1 was reversed (Fig. 5B). All these results were confirmed by western blot analysis (Fig. 5C). Therefore, while RBM38 was negatively correlated with miR-20a-5p, it showed a positive correlation with CALML3-AS1 (Fig. 5D, *p* < 0.001).

Next, we used CCK8 assay and colony formation assay to identify the influence of RBM38 on BCPAP and K1 cells proliferation, the result revealed that after overexpressing CALML3-AS1, the cell proliferation ability was weakened reduced, but after overexpressing miR-20a-5p or knocking down RBM38 at the same time, the cell proliferation was restored (Fig. 5E, F). Additionally, we found, cell migration and invasion ability were reduced, when CALML3-AS1 was overexpressed. Both the migration and invasion of cells was significantly recovered by overexpressing miR-20a-5p or knocking down RBM38 concomitantly (Fig. 5G, H). However, the apoptosis of BCPAP and K1 cells was changed by changing experiment groups (Fig. 5I). In summary, CALML3-AS1 acts as a ceRNA to inhibit tumor progression via miR-20a-5p/RBM38 axis.

## Discussion

In recent years, research of lncRNA has received more attention, it play an important role in many malignant diseases, such as cardiovascular diseases [20], inflammatory diseases [21], eosinophilic asthma [22] and participate in the progression of cancer [23–25]. LncRNAs can regulate tumorigenesis through multiple molecular mechanisms, such as a novel ceRNA model, for instance, STAT3-mediated acted as a ceRNA to upregulate lncRNA HOXD-AS1 by regulating SOX4 to facilitate liver cancer metastasis [26], lncRNA XLOC_006390 promotes cervical cancer progress as a ceRNA sponge miR-331-3p and miR-338-3p [27]. CALML3-AS1 is one of the typical lncRNA, studies have revealed that CALML3-AS1 plays a crucial role in bladder cancer, and such cancer-promoting potential also existed in cervical cancer [28, 29], but the research in PTC is not clear. In this article, we investigated the biological function and the molecular mechanism of CALML3-AS1 in PTC both in vitro and vivo. We first detect the level of CALML3-AS1 expression in the PTC cells and paracarcinoma, and focused on exploring the role of CALML3-AS1 in thyroid cancer at the cytological level subsequently. qRT-PCR analysis found that CALML3-AS1 is significantly down-regulated in PTC tissues and cells. To further investigate the influences of CALML3-AS1 dysregulation on PTC cells activities, we artificially change its expression to study mechanism in BCPAP and K1 cell lines. According to the results of functional assays, we confirmed that overexpression of CALML3-AS1 can inhibit cell proliferation, migration, invasion and induce cell apoptosis. Therefore, CALML3-AS1 exhibited suppress property in PTC and be able to become a potential target for treating PTC.

Previous research has shown that lncRNA can sponge miRNA regulate tumorigenesis. For example, curcumin inhibits proliferation and invasion of prostate cancer cells by ceRNA effect of miR-145 and lncRNA-ROR [30], squamous cell carcinoma of tongue was integrated analysis by lncRNA-miRNA-mRNA ceRNA network [31]. Here, we hypothesized that CALML3-AS1 might act as a ceRNA by sponging a certain miRNA. Firstly, bioinformatics analysis, luciferase reporter assay, and pull-down assay were conducted to find the target miRNA of CALML3-AS1 in PTC cells. The results showed that miR-20a-5p is a target of CALML3-AS1, the negative correlation between them was analyzed and identified with Pearson correlation analysis and proved that CALML3-AS1 can negatively regulate miR-20a-5p. miR-20a-5p was recently identified as a cancer promoter, which can promote triple-negative breast cancer cells growth via targeting RUNX3 [32, 33] and promote colorectal cancer invasion by downregulating Smad4 [34]. In this study, we examined the expression pattern of miR-20a-5p in PTC tissues and cells. As a result, miR-20a-5p was overexpressed in PTC tissues and cell lines. Subsequently, RBM38 was found to be the target of miR-20a-5p and the results of qRT-PCR analysis showed that RBM38 was significantly down-expressed in PTC cells.

In previous studies, it was confirmed that RBM38 plays a tumor suppressor role in cancer [35], such as gastric cancer [36], breast cancer [37]. Therefore, we further verified the molecular mechanism of CALML3-AS1 through miR-20a-5p and RBM38 on cancer regulation. To verify our research, we transfected miR-20a-5p or RBM38 while overexpressing CALML3-AS1 in PTC cell lines, and it was found that overexpression of CALML3-AS1 and up-regulation of miR-20a-5p would lead to a decrease in RBM38, but its level rose after be transfected.

In summary, it was confirmed that CALML3-AS1 suppresses papillary thyroid cancer progression via sponging miR-20a-5p/RBM38 axis. Our experimental findings might provide the potential therapeutic target for PTC.

## Conclusion

We concluded that CALML3-AS1 suppresses PTC progression by downregulating the RBM38 expression. Our results for the first time demonstrated that CALML3-AS1 inhibits PTC tumorigenesis as a ceRNA through sponge miR-20a-5p to regulate expression of RBM38. This work helps us to better understand the mechanism of LncRNAs in PTC progression and provide a novel biomarker for PTC treatment.

## Supplementary Information

**Additional file 1.** Supplementary WB-1 for Fig. 4C (upper panel RBM38 and GAPDH in BCPAP cells). Supplementary WB-2 for Fig. 4C (upper panel RBM38 and GAPDH in K1 cells). Supplementary WB-3 for Fig. 5C (upper panel RBM38 and GAPDH in BCPAP cells). Supplementary WB-4 for Fig. 5C (upper panel RBM38 and GAPDH in K1 cells).

## Data Availability

All supporting data of this work, which are not available in public because of the ethical restrictions are available from the corresponding author upon request.
