# Supplementary material for: LncRNA CALML3-AS1 suppresses papillary thyroid cancer progression via sponging miR-20a-5p/RBM38 axis
Source: BMC Cancer. 2022 Mar 29;22:344. doi: 10.1186/s12885-022-09360-3 (PMC8966157; doi:10.1186/s12885-022-09360-3)
Supplement: Supplementary file 1 — Additional file 1. Supplementary WB-1 for Fig. 4C (upper panel RBM38 and GAPDH in BCPAP cells). Supplementary WB-2 for Fig. 4C (upper panel RBM38 and GAPDH in K1 cells). Supplementary WB-3 for Fig. 5C (upper panel RBM38 and GAPDH in BCPAP cells). Supplementary WB-4 for Fig. 5C (upper panel RBM38 and GAPDH in K1 cells). [file 12885_2022_9360_MOESM1_ESM.pptx]

## Slide 1
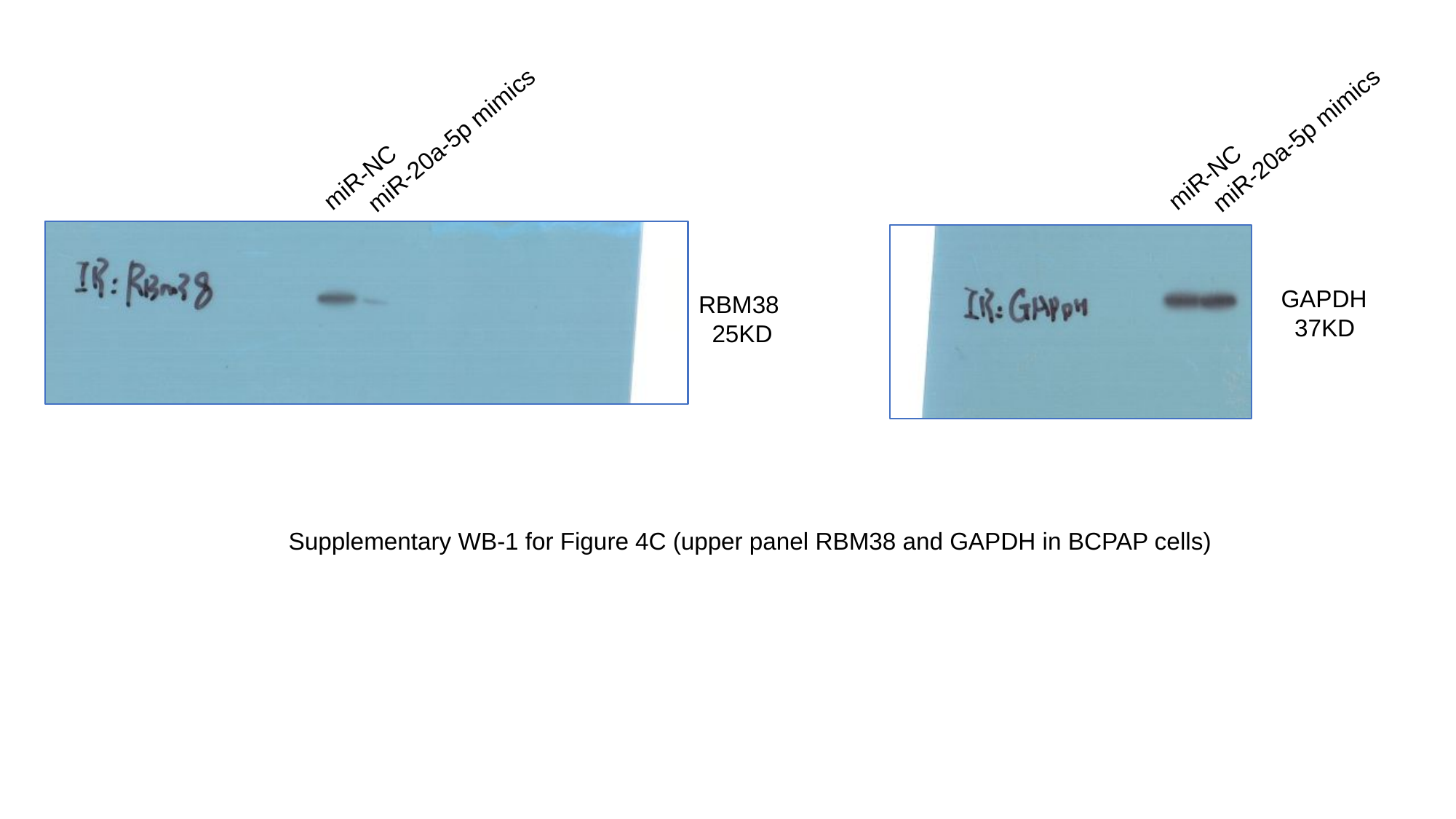

miR-20a-5p mimics
miR-20a-5p mimics
miR-NC
miR-NC
GAPDH
 37KD
RBM38
 25KD
Supplementary WB-1 for Figure 4C (upper panel RBM38 and GAPDH in BCPAP cells)

## Slide 2
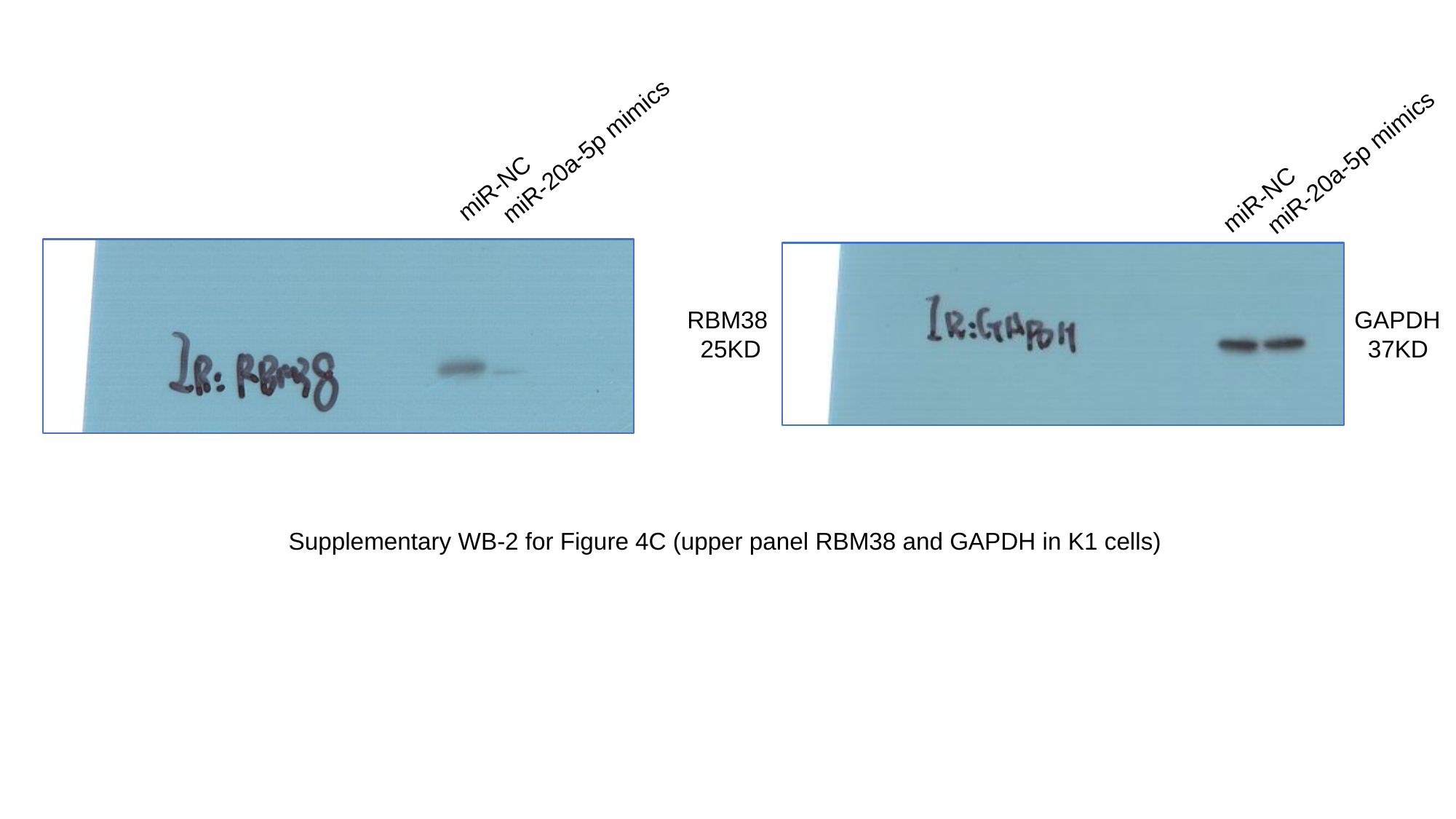

miR-20a-5p mimics
miR-20a-5p mimics
miR-NC
miR-NC
GAPDH
 37KD
RBM38
 25KD
Supplementary WB-2 for Figure 4C (upper panel RBM38 and GAPDH in K1 cells)

## Slide 3
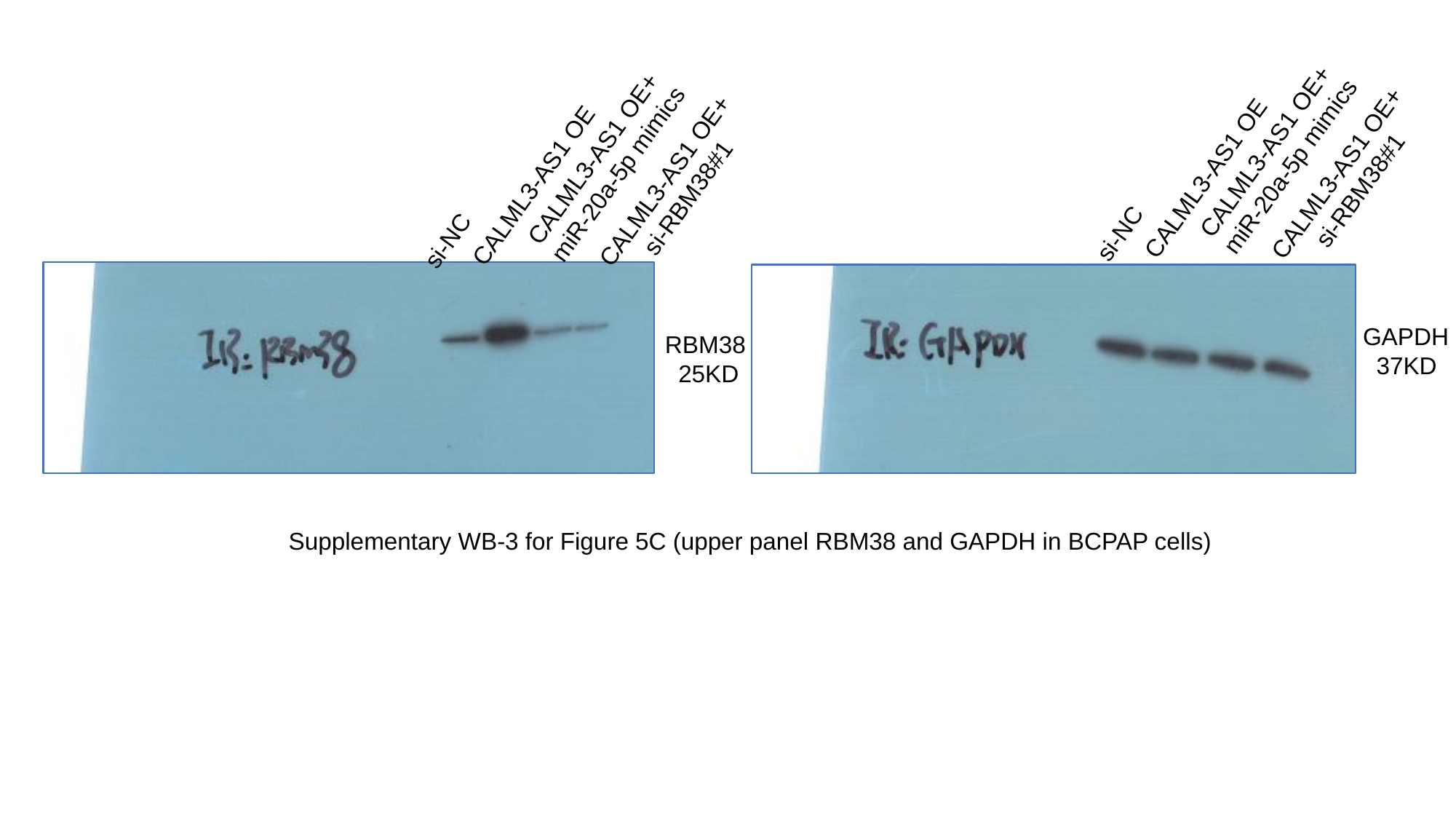

CALML3-AS1 OE+
miR-20a-5p mimics
CALML3-AS1 OE
CALML3-AS1 OE+
miR-20a-5p mimics
CALML3-AS1 OE
CALML3-AS1 OE+
si-RBM38#1
CALML3-AS1 OE+
si-RBM38#1
si-NC
si-NC
GAPDH
 37KD
RBM38
 25KD
Supplementary WB-3 for Figure 5C (upper panel RBM38 and GAPDH in BCPAP cells)

## Slide 4
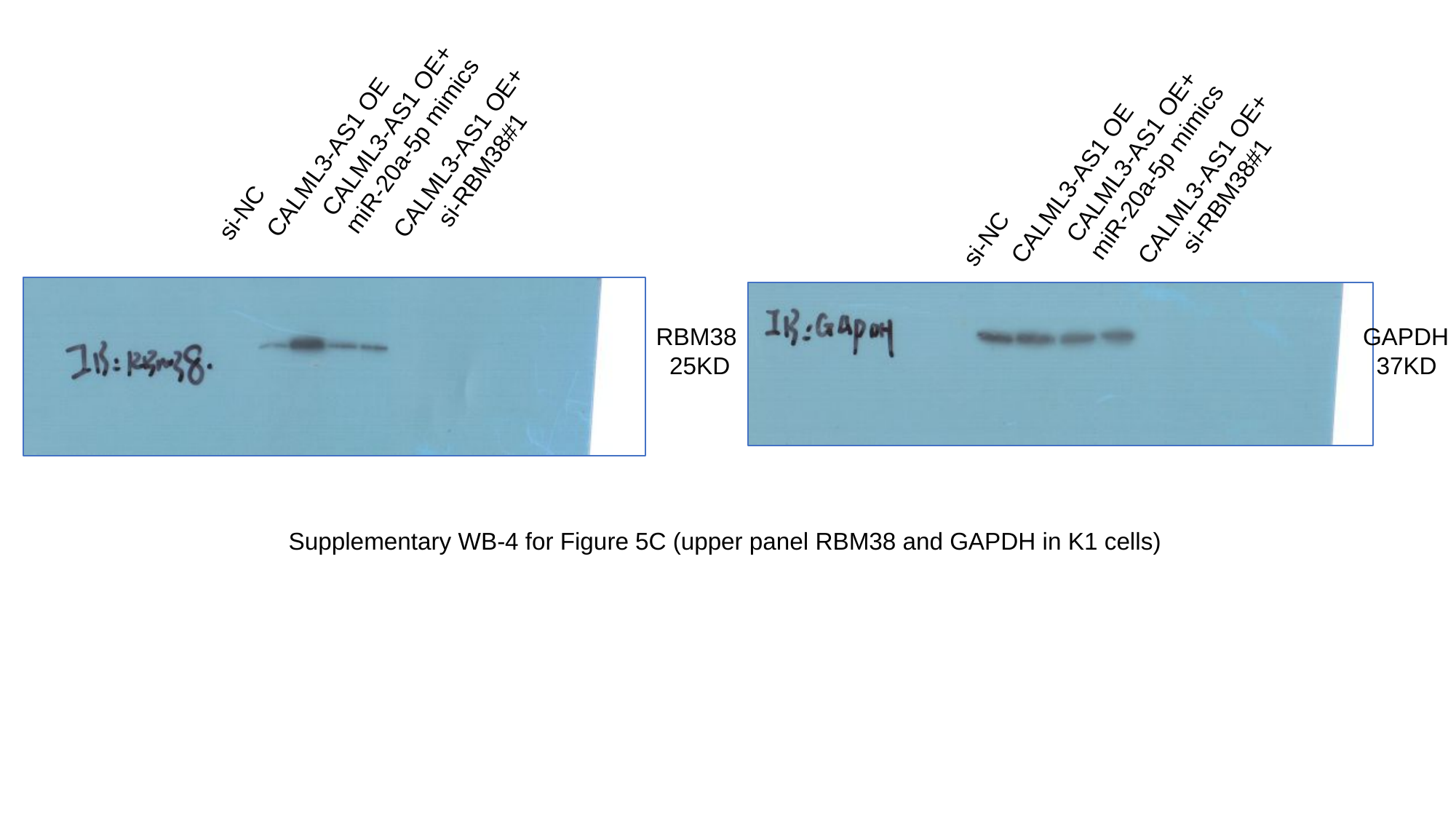

CALML3-AS1 OE+
miR-20a-5p mimics
CALML3-AS1 OE
CALML3-AS1 OE+
miR-20a-5p mimics
CALML3-AS1 OE
CALML3-AS1 OE+
si-RBM38#1
CALML3-AS1 OE+
si-RBM38#1
si-NC
si-NC
RBM38
 25KD
GAPDH
 37KD
Supplementary WB-4 for Figure 5C (upper panel RBM38 and GAPDH in K1 cells)
